# TSPO Ligands Promote Cholesterol Efflux and Suppress Oxidative Stress and Inflammation in Choroidal Endothelial Cells

**DOI:** 10.3390/ijms19123740

**Published:** 2018-11-24

**Authors:** Lincoln Biswas, Fahad Farhan, James Reilly, Chris Bartholomew, Xinhua Shu

**Affiliations:** 1 Department of Biological and Biomedical Sciences, Glasgow Caledonian University, Glasgow G4 0BA, UK; Lincoln.Biswas@gcu.ac.uk (L.B.); Fahad.Farhan@gcu.ac.uk (F.F.); J.Reilly@gcu.ac.uk (J.R.); C.Bartholomew@gcu.ac.uk (C.B.); 2 Department of Vision Science, Glasgow Caledonian University, Glasgow G4 0BA, UK

**Keywords:** TSPO, cholesterol efflux, oxidative stress, inflammation, choroidal endothelial cells, age-related macular degeneration

## Abstract

Choroidal endothelial cells supply oxygen and nutrients to retinal pigment epithelial (RPE) cells and photoreceptors, recycle metabolites, and dispose of metabolic waste through the choroidal blood circulation. Death of the endothelial cells of the choroid may cause abnormal deposits including unesterified and esterified cholesterol beneath RPE cells and within Bruch’s membrane that contribute to the progression of age-related macular degeneration (AMD), the most prevalent cause of blindness in older people. Translocator protein (TSPO) is a cholesterol-binding protein that is involved in mitochondrial cholesterol transport and other cellular functions. We have investigated the role of TSPO in choroidal endothelial cells. Immunocytochemistry showed that TSPO was localized to the mitochondria of choroidal endothelial cells. Choroidal endothelial cells exposed to TSPO ligands (Etifoxine or XBD-173) had significantly increased cholesterol efflux, higher expression of cholesterol homeostasis genes (*LXRα*, *CYP27A1*, *CYP46A1*, *ABCA1* and *ABCG1*), and reduced biosynthesis of cholesterol and phospholipids from [^14^C]acetate, when compared to untreated controls. Treatment with TSPO ligands also resulted in reduced production of reactive oxygen species (ROS), increased antioxidant capacity, and reduced release of pro-inflammatory cytokines (IL-1β, IL-6, TNF-α and VEGF) induced by oxidized LDL. These data suggest TSPO ligands may offer promise for the treatment of AMD.

## 1. Introduction

Age-related macular degeneration (AMD) is the major cause of visual dysfunction in the elderly population. Due to population ageing, it is predicted that the number of AMD patients will be increased from 196 million in 2020 to 288 million by 2040 [1]. AMD is classed as two types: the dry form with geographic atrophy and the wet form with neovascularization. Pathological characterization of retinas from AMD patients demonstrated that photoreceptors, retinal pigment epithelial (RPE) cells and choroidal endothelial cells were affected and had degenerated [2]. Choroidal endothelial cells were markedly lost in AMD patients, the loss occurring earlier than that of RPE cells [2]. Another study also suggested early degeneration of choroidal endothelial cells in dry AMD, showing significantly decreased expression of endothelium-associated genes in RPE/choroid [3]. The choriocapillaris (CC) provides oxygen and nutrients for RPE cells and for photoreceptors and clears metabolic waste from these cells via the systemic circulation [4]. Previous studies showed that CC density was significant lower in AMD patients compared to control groups of the same age [2] and that loss of CC was positively correlated with drusen formation in early AMD [5]. An early study in rabbits demonstrated that RPE cells were required for modulating CC structure and function [6]. However, analysis of AMD patient samples showed that, in dry AMD, the CC survived even though RPE cells were completely lost, while in wet AMD the CC underwent marked degeneration [2,7]. Early loss of choroidal endothelial cells and dysfunction of CC are believed to result in a defective supply of nutrients and oxygen to RPE and photoreceptors, while more importantly compromising the recycling of metabolites and the disposal of metabolic waste, thus leading to the formation of deposits (e.g., drusen) within Bruch’s membrane and beneath the RPE [7].

Translocator protein (TSPO, 18 kDa) is a mitochondrial outer membrane protein, first identified as a benzodiazepine receptor [8]. Rat TSPO can rescue the phenotype presented in *Rhodobacter sphaeroides* with deletion of TSPO [9], indicating functional conservation in evolution. TSPO expresses at a high level in a variety of tissues, particularly so in steroidogenic tissues. TSPO has five transmembrane (TM) domains, in which TM5 holds putative cholesterol-binding sites [10]. TSPO also can bind different types of ligands, which mediates its function. The major function of TSPO is transporting cholesterol from the mitochondrial outer membrane (MOM) to the mitochondrial inner membrane (MIM) where cholesterol is metabolized into pregnenolone by Cyp11A in steroid-producing cells or into oxysterols by CYP27A1 in non-steroidogenic cells [11]. However global or conditional *Tspo* knock-out mouse models have exhibited divergent phenotypes (embryonic lethal, or defect in steroidogenesis, or no effect on steroidogenesis), possibly due to background genetic differences between strains of those KO mice [12,13,14,15,16]. TSPO also has important roles to play in other cellular processes: it interacts with the adenine nucleotide transport (ANT) and the voltage-dependent anion channel (VDAC) [17], which are core components of the mitochondrial permeability transition pore; TSPO also regulates production of reactive oxygen species (ROS) and mediates apoptosis [18]. TSPO expression is markedly elevated in activated microglia in an injured brain. It is also upregulated in neurodegenerative conditions, including amyotrophic lateral sclerosis, multiple sclerosis, Alzheimer’s disease, Huntington’s disease and Parkinson’s disease [19]. Both in injured and degenerating mouse retinas, TSPO expression is also upregulated in activated microglia [20,21]. Knockdown of TSPO in BV2 microglia caused significantly increased production of ROS and TNF-α, suggesting thatTSPO negatively regulates microglial activation [20].

Recently we found TSPO expressed at a high level in human RPE cell line and in mouse RPE [16]. Treatment with TSPO ligands (Etifoxine, XBD173 and FGIN-1-27) caused a rise in cholesterol efflux, decreased lipogenesis, reduced cellular phospholipids and total cholesterol, and upregulation of cholesterol metabolism and transporter genes in RPE cells. Loss of TSPO caused increased lipid accumulation and production of ROS and pro-inflammatory cytokines [22]. Prior to the present study, there have been no investigations of TSPO function in choroidal endothelial cells. We found that treatment with TSPO ligands resulted in increased cholesterol efflux to high-density lipoprotein (HDL), reduced intracellular lipid accumulation and decreased production of ROS and secretion of cytokines induced by oxidized low-density lipoprotein (oxLDL) in choroidal endothelial cells. Our observations suggest that TSPO ligands have therapeutic potential for AMD.

## 2. Results

### 2.1. TSPO Ligands Increase Cholesterol Efflux and Upregulate Cholesterol Metabolism and Transporter Genes

**Figure 1:**
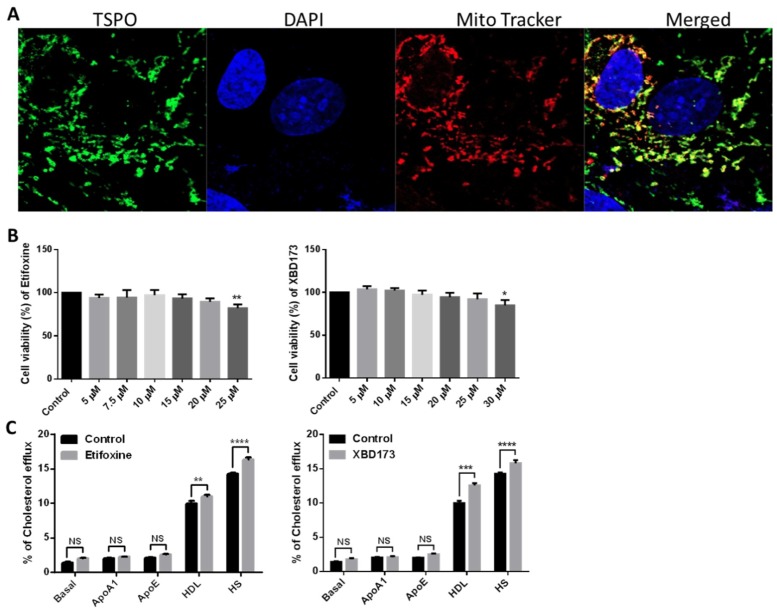
(**A**) The expression and co-localisation of translocator protein (TSPO) and mitochondria in RF/6A cells. RF/6A cells were stained with Mito-tracker (a marker for functional mitochondria) following cell culture and then fixed with cold methanol. Subsequently, the slides were treated with anti-TSPO antibody. Images captured confocally show TSPO (Green), DAPI (Blue), mitochondria (Red), while the Merged image shows co-localisation of TSPO and mitochondria (630×). (**B**) Toxic effects of TSPO ligands Etifoxine and XBD173 on RF/6A cells. RF/6A cells were treated with different concentrations of Etifoxine for 24 h and assayed: a decline in cell viability at the concentration of 25 µM was noted compared to the untreated controls; similarly, 30 µM XBD173 had a significant toxic effect compared to the control. (**C**) Efflux of cholesterol initiated by acceptors (APOA1, ApoE, HDL and HS) and incubated with TSPO ligands Etifoxine (20 µM) and XBD173 (25 µM) was studied in RF6A cells. The percentage of [^3^H]cholesterol was calculated in the media and in the cells. Data for (**B)** and (**C**) are shown as mean ± SD. Statistical comparisons were done using One-way ANOVA (**B**) and Two-way ANOVA (**C**), followed by Bonferroni test. Each experiment was performed in triplicate and repeated twice. NS: non-significant, * *p* ≤ 0.05, ** *p* < 0.01, *** *p* < 0.001 and **** *p* < 0.0001.

**Figure 2:**
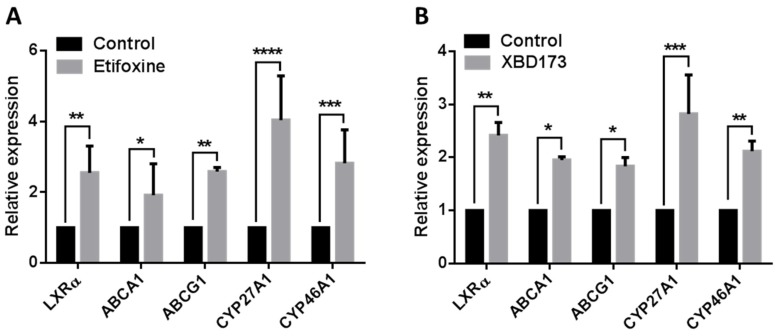
The effects of TSPO ligands on the expression of cholesterol efflux genes and metabolism genes in RF6A cells treated with Etifoxine (**A**) or XBD173 (**B**) were quantified by qRT-PCR. The RF/6A cells were cultured and treated with TSPO ligands Etifoxine (20 µM) and XBD173 (25 µM) and with 0.1% DMSO as a control for 24 h. All data were collected and analysed relative expression of β-actin and presented as mean ± SD. Statistical comparisons were done using Two-way ANOVA followed by Bonferroni test. NS: non-significant, * *p* ≤ 0.05; ** *p* ≤ 0.01; *** *p* < 0.001 and *** *p* ≤ 0.001.

Previous studies reported that TSPO is expressed in a wide range of cells, including umbilical vein endothelial cells, microglia, macrophages, astrocytes, fibroblasts and RPE cells [20,21,22,23,24,25,26,27,28]. We investigated TSPO expression in choroidal endothelial cells by immunostaining using a specific TSPO antibody and found that TSPO co-localized to mitochondria with Mito Tracker, a marker of functional mitochondria (Figure 1A). TSPO ligands have been shown to increase cholesterol efflux from fibroblasts, macrophages and RPE [22,23,24]. We determined the potential toxic effects of TSPO ligands by treating primate choroidal endothelial cells (RF/6A) with either Etifoxine (0, 5, 7.5, 10, 15, 20 and 25 µM) or with XBD173 (0, 5, 10, 15, 20, 25 and 30 µM) and measured cell viability after 24 h exposure using MTT assay. RF/6A cells tolerated Etifoxine treatment at the concentrations of 5–20 µM but demonstrated a notable decrease in cell viability at the concentration of 25 µM; similarly, RF/6A cells tolerated XBD173 treatment at doses of 5–25 µM but exhibited significantly decreased cell viability at the dose of 30 µM (Figure 1B). Consequently, we chose Etifoxine at concentration of 20 µM and XBD173 at concentration of 25 µM for subsequent treatments. We treated RF/6A with Etifoxine (20 µM) and XBD173 (25 µM) for 24 h and found that exposure to both ligands caused a significant increase in cholesterol efflux to HDL and human serum (Figure 1C). We further investigated if TSPO ligands affect expression of genes involved in cholesterol homeostasis. After RF/6A cells were treated with TSPO ligands for 24 h, expression of the *NR1H3* gene (coded for oxysterol receptor LXRα), transporter genes (*ABCA1* and *ABCG1*) and metabolism genes (*CYP27A1* and *CYP46A1*) was markedly increased in ligand-treated RF/6A cells (Figure 2).

### 2.2. TSPO Ligands Regulate Lipogenesis in Choroidal Endothelial Cells

**Figure 3:**
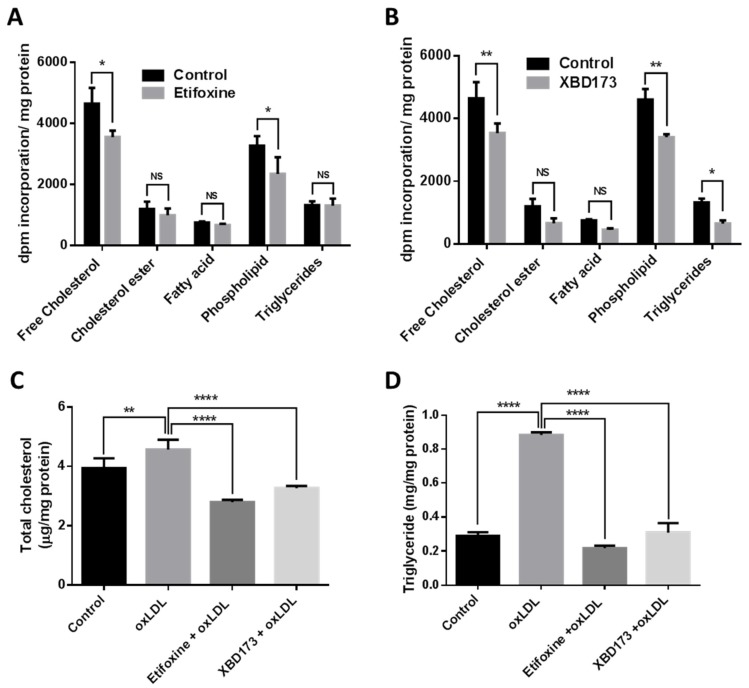
TSPO ligands modified the lipid phenotypes in RF/6A cells. The effect of TSPO ligands ((**A**): Etifoxine, 20 µM; (**B**): XBD173, 25 µM) were studied on disintegrations per minute (dpm) incorporation/mg total protein of [^14^C]acetate (1 µCi/mL) into free cholesterol, cholesteryl ester, fatty acid, phospholipid and triglycerides compared with the 0.1% vehicle control. These TSPO ligands also were applied to RF/6A cells to quantify the total cellular cholesterol (**C**) after the excitation of oxLDL and with their vehicle control. Similarly, it was applied to quantify triglycerides level. All data was represented as mean ± SD and analysed by Two-way ANOVA (**A**,**B**) and one-way ANOVA (**C**,**D**) followed by Bonferroni test. Three independent experiments were performed and the value of statistical were pointed as * *p* ≤ 0.05; ** *p* < 0.01, **** *p* < 0.0001. NS: non-significant.

We examined the effect of TSPO ligands on lipogenesis in RF/6A cells by quantification of synthesized cholesteryl, fatty acid, triglycerides and phospholipid. We found that incorporation of [^14^C]acetate into the free cholesterol pool was significantly decreased by 23.6% in Etifoxine-treated cells and by 23.9% in XBD173-treated cells, compared to control cells; incorporation of [^14^C]acetate into phospholipid was also significantly decreased by 28% in Etifoxine-treated cells and by 26% in XBD173-treated cells, compared to control cells (Figure 3A,B). However, incorporation of [^14^C]acetate into cholesterol ester and fatty acid was not significantly changed in Etifoxine-treated or XBD173-treated cells when compared to control cells; although incorporation of [^14^C]acetate into triglycerides was not significantly changed in Etifoxine-treated cells, it was significantly reduced in XBD173-treated cells (Figure 3A,B), compared to control cells. We also quantified total cholesterol and triglyceride in control cells and in cells incubated with oxLDL, or with oxLDL and TSPO ligand. OxLDL-treated cells had a 13.7% increase in cholesterol and 67% increase in triglyceride when compared to control cells (Figure 3C,D). Total cholesterol was significantly decreased by 38% in Etifoxine-treated cells and by 28% in XBD173-treated cells when compared to cells treated with oxLDL only (Figure 3C); triglyceride was also markedly decreased by 75.1% in Etifoxine-treated cells and by 65% in XBD173-treated cells when compared to cells treated solely with oxLDL (Figure 3D).

**Figure 4:**
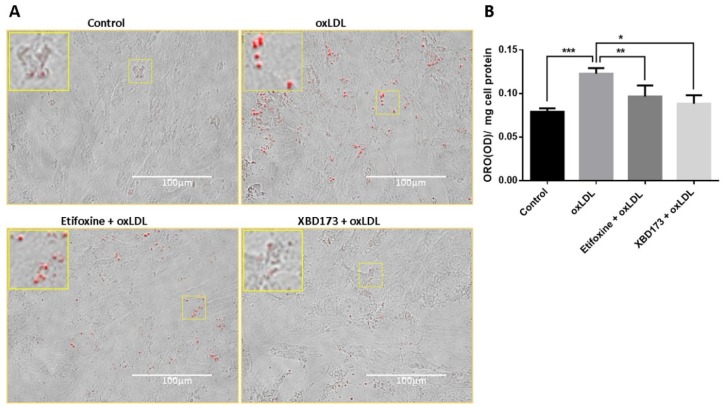
Lipid droplets were detected in RF6A cells by Oil Red O (ORO) staining after treatment with oxLDL and with oxLDL + TSPO ligands. The oxLDL-treated cells were compared to cells treated with Etifoxine (20 µM) + oxLDL or with XBD173 (25 µM) + oxLDL and to vehicle-treated controls. The cells were then fixed with 10% formalin and stained with ORO and images were captured by EVOS microscopy (**A**). Inserts show a 2.5× magnification of the selected regions. The lipid droplets were eluted with 100% isopropanol and optical density (OD) at 520 nm was measured using a plate absorbance reader. The OD was normalised with respect to total cell protein. The data were analysed using one-way ANOVA followed by Bonferroni test and presented as mean ± SD (**B**). * *p* ≤ 0.05, ** *p* < 0.01, and *** *p* < 0.001.

Lipid droplets are functional organelles containing mainly cholesterol esters and triglycerides. Lipid droplets not only manage lipid storage but also mediate a variety of physiological functions such as protein quality control and immune response [29]. We examined lipid droplets in control and treated cells using oil red O (ORO) staining. We found that there was a significantly increased number of lipid droplets in oxLDL treated cells compared to untreated control cells. Treatment with TSPO ligand, Etifoxine or XBD173, notably decreased the quantity of lipid droplets compared to cells treated solely with oxLDL (Figure 4).

### 2.3. TSPO Ligands Supress ROS Production and Increased Antioxidant Capacity

**Figure 5:**
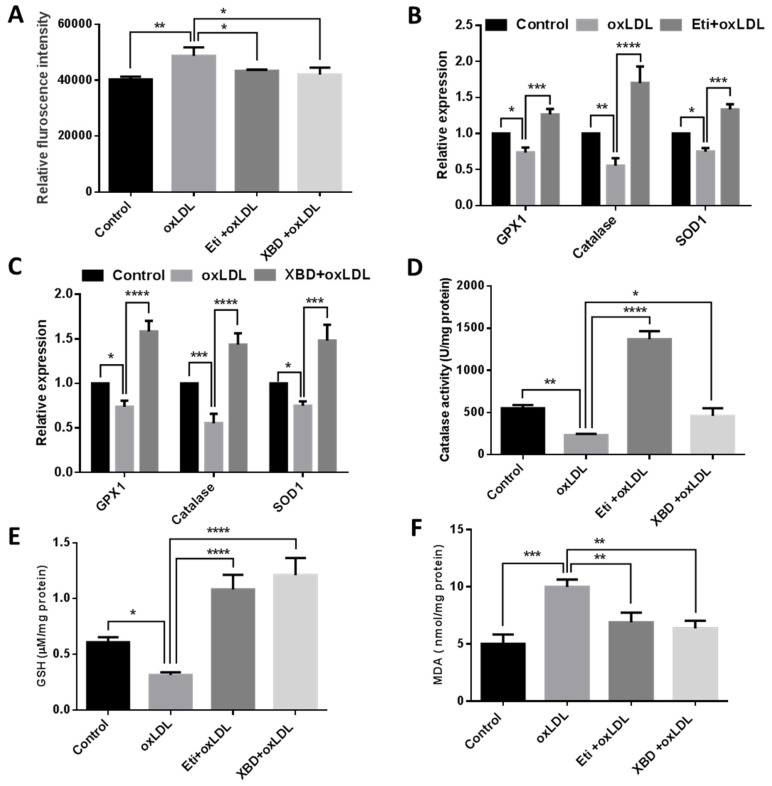
(**A**) TSPO ligands reduced the oxLDL-generated intra-cellular reactive oxygen species (ROS) level in RF6A cells. ROS levels in cells following treatment with oxLDL were compared to cells treated with Etifoxine (20 µM) + oxLDL, or with XBD173 (25 µM) + oxLDL for 24 h, and to vehicle-treated control cells. The mRNA expressions of oxidative genes (*GPX1*, *Catalase* and *SOD1*) were down-regulated following treatment with oxLDL compared to controls. Treatment with Etifoxine (**B**) or XBD173 (**C**) resulted in recovery of gene expression. The protein activity of Catalse (**D**), GSH (**E**) and MDA (**F**) were measured following treatment with oxLDL, with Etifoxine + oxLDL, and with XBD173 + oxLDL. The experiments were performed three times. All data are shown as mean ± SD and analysed using one-way ANOVA (**A**,**D**–**F**) and Two-way ANOVA (**B**,**C**), followed by the Bonferroni test. * *p* ≤ 0.05, ** *p* < 0.01, *** *p* < 0.001 and **** *p* < 0.0001.

Previous studies have demonstrated that TSPO ligands can inhibit ROS generation in different types of cells [30,31,32]. It is well documented that oxLDL can induce ROS generation and cause oxidative damage [33]; accordingly, we treated RF/6A cells with oxLDL to induce ROS generation. RF/6A cells exposed to oxLDL had a significant increase in ROS production compared to untreated control cells. Co-treatment with Etifoxine or XBD173 resulted in notably decreased ROS production (Figure 5A). As ROS generation causes imbalance of antioxidant defence, we investigated antioxidant gene expression in RF/6A cells. OxLDL treatment caused a significant decrease in *GPX1*, *Catalase* and *SOD1* mRNA levels by, respectively, 26%, 45% and 25% (Figure 5B,C), compared to untreated control cells. Co-treatment with Etifoxine significantly increased expression of *GPX1*, *Catalase* and *SOD1* by, respectively, 52%, 115% and 58%, compared to cells treated solely with oxLDL (Figure 5B); similarly, expression of *GPX1*, *Catalase* and *SOD1* was also notably increased by, respectively, 84%, 88% and 73% in cells co-exposed to oxLDL and XBD173, compared to cells treated with oxLDL alone (Figure 5C).

We also examined the effect of TSPO ligands on Catalase activity and the levels of glutathione (GSH) and malondialdehyde (MDA). Catalase activity was significantly decreased by 58% in oxLDL-treated cells when compared to untreated cells; co-treatment with Etifoxine or XBD173 led to notable increases in Catalase activity by 492% and 98% respectively, when compared to cells treated solely with oxLDL (Figure 5D). GSH, an antioxidant that protects cells from ROS-induced damage, was significantly decreased by 48.5% in oxLDL-treated cells when compared to untreated cells. GSH was markedly increased by 245% and 286%, respectively, in oxLDL-treated cells co-exposed to Etifoxine or XBD173 when compared to cells treated with oxLDL alone (Figure 5E). However, MDA, a lipid peroxidation marker, was significantly increased by 49.9% in oxLDL treated cells when compared to untreated controls; co-treatment with Etifoxine or XBD173 resulted in notable decreases in MDA levels by 31% and 36.29% respectively, compared to cells treated solely with oxLDL (Figure 5F).

### 2.4. TSPO Ligands Decrease Production of Pro-Inflammatory Cytokines

**Figure 6:**
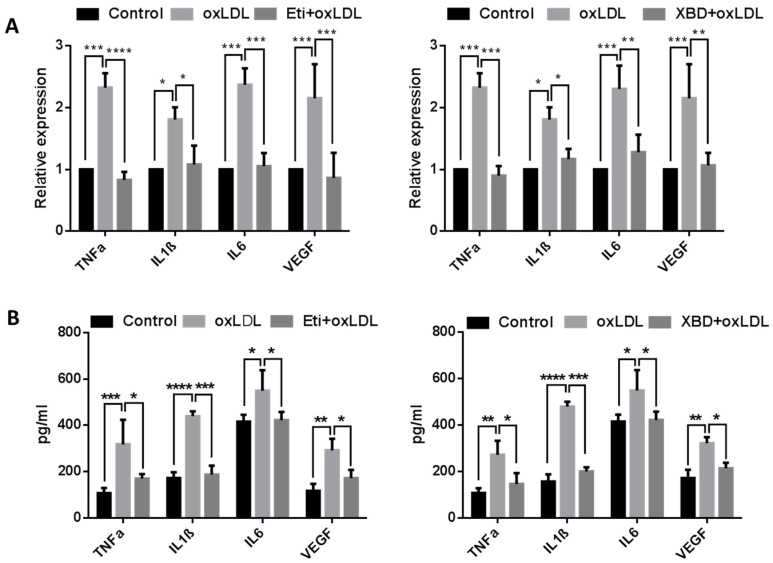
The expression of inflammation genes was quantified in RF/6A cells following treatment with oxLDL and TSPO ligands. (**A**) mRNA expression of *TNF-α*, *IL1-β*, *IL-6* and *VEGF* relative to *β-ACTIN* was measured following treatment with oxLDL and compared to that seen in cells treated additionally with Etifoxine (20 µM) or XBD173 (25 µM) for 24 h and in vehicle-treated control cells. (**B**) Following the same treatment, the protein concentrations of these genes were measured by ELISA. Data are represented as mean ± SD and were analysed by Two-way ANOVA followed by Bonferroni multiple comparison test. * *p* < 0.05, ** *p* < 0.01, *** *p* < 0.001 and **** *p* < 0.0001.

OxLDL has been shown to induce secretion of pro-inflammatory cytokines, such as IL-1β and TNF-α, in vascular cells [34,35]. We investigated the effects of TSPO ligands on production of pro-inflammatory cytokines induced by oxLDL in choroidal endothelial cells. Firstly, we did quantitative real-time polymerase chain reaction (qRT-PCR) to detect mRNA of *TNFα*, *IL-1β*, *IL-6* and *VEGF* genes and found expression of all examined cytokine genes was significantly increased in oxLDL-treated cells when compared to untreated controls; co-treatment with Etifoxine or XBD173 notably downregulated expression of these genes (Figure 6A). We also used ELISA to measure secreted TNF-α, IL-1β, IL-6 and VEGF and found that cells treated with oxLDL had a significant increase in levels of these cytokines when compared to untreated cells. Similarly, co-exposure to Etifoxine or XBD173 led to notable decrease in production of these cytokines when compared to cells treated with oxLDL alone (Figure 6B).

## 3. Discussion

The major clinical feature of AMD is formation of abnormal extracellular deposits known as drusen [36]. Pathogenic analyses of AMD patient eyes demonstrated unesterified and esterified cholesterol were prominent in drusen [37], implicating abnormal cholesterol homeostasis in the condition. It is proposed that excess cholesterol from RPE is recycled back to the liver for storage or excretion through reverse cholesterol transport (RCT) [22,38,39]. Under physiological conditions RPE cells export cholesterol to Bruch’s membrane (BrM) via ABCA1; cholesterol then crosses BrM and enters the choroidal circulation [40]. However, the role of choroidal endothelial cells involved in the RCT in RPE/BrM/choroid has not been studied, though the RCT in vascular endothelial cells has been well investigated [41]. The current study demonstrated that TSPO ligands promoted cholesterol efflux, decreased accumulation of oxidized LDL, increased antioxidative capacity and inhibited inflammation in choroidal endothelial cells.

TSPO directly transports cholesterol from the MOM to the MIM, and TSPO ligands have been shown to promote mitochondrial cholesterol movement [11]. However, the concept of TSPO-mediated steroid biosynthesis faces challenge, as deletion of TSPO in mice and in MA-10 Leydig cells presented inconsistent phenotypes [12,13,14,15,16,42,43]. In non-steroid-producing cells (a human macrophage cell line), Taylor et al. reported that overexpression of TSPO increased cholesterol efflux and upregulated cholesterol-efflux-associated genes including *NR1H3*, *ABCA1*, *ABCG4* and *APOE;* conversely, depletion of TSPO by siRNA knockdown caused the opposite effect. Treatment with TSPO ligands (PK11195, FGN-1-27 and flunitrazepem) significantly increased cholesterol efflux [24]. In human RPE (ARPE-19) cells, TSPO ligands (FGIN-1-27, XBD173 and Etifoxine) enhanced cholesterol efflux and increased expression of cholesterol transport and metabolism genes, including *NR1H3*, *ABCA1*, *ABCG1*, *CYP27A1* and *CYP46A1* [22]. In the present study, TSPO ligands (XBD173, 25 µM and Etifoxine, 20 µM) enhanced cholesterol efflux and upregulated expression of cholesterol-efflux-related genes in choroidal endothelial cells (Figure 1C and Figure 2). Our previous work also used Etifoxine and XBD173 at same concentration to treat RPE cells and found the effect of both ligands on cholesterol efflux was TSPO-dependent [22], though early reports suggested Etifoxine also binds to GABA_A_Rs on β subunits [44]. Interesting Costa et al. recently reported that the neurosteroidogenic ability of both Etifoxine and XBD173 is dependent on residence time at the binding site rather than their binding affinities [45,46]. It also worth noting that treatment with TSPO ligands resulted in decreased lipogenesis in macrophages, RPE and choroidal endothelial cells, and that free cholesterol was significantly lower in TSPO-ligand-treated RPE and choroidal endothelial cells when compared to untreated controls (Refs. [22] and [24], Figure 3A,B). Cholesterol accumulation is thought to promote inflammation in macrophages that is associated certain chronic metabolic diseases such as atherosclerosis and obesity [47]. However, whether decreased free cholesterol in TSPO-ligand-exposed choroidal endothelial cells will reduce intracellular cholesterol accumulation and repress inflammation needs further investigation. 

TSPO ligands have demonstrated anti-oxidative-stress and anti-inflammation capacity against different types of stress in vitro and in vivo [48,49]. Early studies reported that TSPO ligands (SSR180575 and Ro5-4864) protected against mitochondrial dysfunction and prevented cell death caused by H_2_O_2_ exposure in COS7, HEK293, H9C2 and human renal proximal tubule epithelial cells through inhibition of cytochrome c release, increase of mitochondrial membrane potential, and decrease of caspase 3 activation [50,51]. SSR180575 also showed protection against cardiac and renal cell death following ischemia-reperfusion (I-R) induced injury [49,50]. Similarly, TSPO ligands Ro5-4864 and TRO40303 preserved cardiac function against oxidative damage associated with I-R injury [52,53,54]. Baez et al. recently demonstrated that TSPO ligand Ro5-4864 reduced production of free radicals and helped to maintain mitochondrial function in astrocytic glioblastoma T89G cells under metabolic damage [55]. TSPO ligand PIGAS also displayed antioxidative capacity in astrocytic C6 glioma cells under l-buthionine-(*S*,*R*)-sulfoximine (BSO) induced toxicity, possibly through stimulation of neurosteroid synthesis [56]. In addition, TTN, PK11195 and Ro5-4864 decreased LPS-induced ROS production in microglia cells [20]. TSPO ligands also showed anti-inflammatory effects in astrocytic, microglial and vascular endothelial cells. PIGAS suppressed LPS/IFNγ-induced iNOS and COX-2 expression in astrocytic C6 glioma cells [56]. Etifoxine also decreased LPS-induced release of IL-1β and IL-6 in rat primary astrocytes [57]. PK11195 down-regulated expression of Cox-2 in human microglia exposed to LPS [58] and inhibited nitric oxide production in rat primary microglia challenged with LPS [59]. PK11195, TTN and Ro5-4864 also downregulated TNF-α expression in human and rodent microglia incubated with LPS [19,57]. LPS-challenged microglial cells (BV-2) treated with XBD173 had significantly decreased expression of pro-inflammatory genes including *CCL-2*, *IL-6* and *iNOS*; XBD173-treated BV-2 cells also demonstrated reduced migratory capacity and proliferation [20]. Endothelial activation is a clinical feature of vascular inflammation and is characterized by the increased expression of cell-surface adhesion factors, e.g., VCAM-1 (vascular cell adhesion molecule-1) [60]. The TSPO ligand midazolam caused inhibition of endothelial activation by lowering VCAM-1 expression in human umbilical vein endothelial cells challenged with TNF-α or the phorbol 12-myristate 13-acetate (PMA) [28,31]. In the present study, for the first time, we demonstrated that TSPO ligands promoted cholesterol efflux (Figure 1C) and upregulated expression of cholesterol metabolism and trafficking genes in choroidal endothelial cells (Figure 2). The two ligands also suppressed ROS production (Figure 5A), increased expression of antioxidant genes (Figure 5B,C) and reduced secretion of pro-inflammatory cytokines including IL-1β, IL-6, TNF-α and VEGF (Figure 6). 

TSPO ligands have shown protective effects in neurodegenerative animal models [61]. Previous studies showed that Ro5-4864 reduced β-amyloid (Aβ) protein deposit and decreased gliosis in the hippocampus of 3xTgAD mice. Aged 3xTgAD mice with Ro5-4864 treatment had decreased anxiety and improved spontaneous alternation behaviour. Combinational treatment with Ro5-4864 and PK-11195 also lowered brain Aβ in nontransgenic young mice [62]. Treatment with Etifoxine or XBD-173 resulted in improved clinical and neuropathological features in rodent multiple sclerosis models through inhibition of inflammation, elevation of neurosteroids and prevention of demyelination [57,63,64]. XBD-173 also showed protection of photoreceptors against light-induced damage [65]. Both Etifoxine and XBD-173 have gone through clinical trials and showed therapeutic effects in patients with anxiety [66,67]. Etifoxine is clinically available and used in more than 42 different countries [67]. Our previous study [22] and current work have demonstrated a functional role of Etifoxine and XBD-173 in RPE and choroidal endothelial cells, which are areas of primary dysfunction in AMD. Consequently, Eitfoxine and XBD-173 may have therapeutic potential for AMD patients.

## 4. Materials and Methods

### 4.1. Cell Viability Assay

RF/6A (ATCC^®^ CRL-1780™) cells were seeded at a density of 30,000 cells/ well in 96-well plates (Greiner Bio One, Stonehouse, UK) with RPMI 1640 medium and cultured in a 5% CO_2_ incubator at 37 °C for 24 h. The cells were treated with different concentrations of Etifoxine and XBD173 (dissolved in dimethyl sulfoxide, 0.1% DMSO) at 5, 7.5, 10, 15, 20, 25 or 30 μM for 24 h, with untreated cells in media with 0.1% DMSO as controls. For the final 2 h of treatment incubation, the cells were treated with 3-(4,5-dimethylthiazol-2-yl)-2,5-diphenyltetrazolium bromide (MTT) solution (0.4 mg/mL and 50 μL/well) (Sigma, Gillingham, UK) according to the manufacturer’s protocols. The optical density (OD) was measured at 570 nm in a microplate reader (Epoch, Biotech, Winooski, VT, USA). The untreated cell viability was taken to represent 100% cell viability. The percentage of cells viability was calculated using the formula: % of viable cells = [(OD of untreated cells − OD of treated cells)/OD of untreated cells] × 100.

### 4.2. Measurement of [^3^H]Cholesterol Efflux

Cholesterol efflux was measured from retinal choroidal endothelial cells that were cultured in RPMI 1640 medium with fetal bovine serum (10%, *v*/*v*) and antibiotics (penicillin and streptomycin, both 50 U/mL). The efflux protocol was adapted from our previous description [22]. Briefly, the RF/6A cells were cultured in 12-well plates at the density of 2 × 10^5^ cells/well. Next, the cells were labelled with [^3^H] cholesterol in 0.1% BSA-added serum-free culture media for 24 h. Cells were exposed to TSPO ligands (Etifoxine, 20 µM or XBD173, 25 µM) and different acceptors: ApoAI (10 µg/mL), ApoE (10 µg/mL), HDL (20 µg/mL) and human serum (1%, *v*/*v*) for 24 h. After the incubation, the labelled [^3^H]cholesterols from the media and from cells were counted on a scintillant counter. Cholesterol efflux to each acceptor was calculated as % efflux = (disintegrations per minute (DPM) media/DPM Media + DPM Cells) × 100.

### 4.3. Lipid Analysis

The incorporated [^14^C]acetate (1 µCi/mL) was measured from the lipid pools of phospholipids, triglycerides, fatty acid, free cholesterol and cholesteryl ester, as previously reported [16]. In brief, the RF/6A cells were cultured on 12 well plates, seeded at a density of 250,000 cells/well. The cells were labelled with [^14^C]acetate (1 µCi/mL). Next day, the cells were treated for 24 h with the TSPO ligands (Etifoxine, 20 µM or XBD173, 25 µM in media containing 0.1% DMSO) and the vehicle (media containing 0.1% DMSO). After 24 h, the cellular lipids were extracted by 3:2 (*v*/*v*) hexanes: isopropanol. The lipid extracts were dried under nitrogen gas and dissolved in isopropanol. The cellular lipids were separated by thin-layer chromatography (TLC) with two mobile phases: phase-I was 60:30:5, (*v*/*v*/*v*) chloroform, methanol and water, while phase-II was 80:20:1.5, (*v*/*v*/*v*) hexane, diethyl ether and acetic acid. The particular lipids were recognised by comigrated standards and incorporation of radiolabel evaluated using scintillation counting.

### 4.4. Lipid Assays

The RF/6A cells were incubated with oxLDL (200 µg/mL) and treated with Etifoxine and XBD173 in media containing 0.1% DMSO or vehicle control (media containing 0.1% DMSO) for 24 h. Following treatment, the mass of total cellular cholesterol and triglycerides were measured using, respectively, the Amplex Red Cholesterol Assay Kit (Thermo Fisher Scientific, Waltham, MA, USA) and EnzyChrom Triglyceride Assay Kit (BioAssay Systems, Hayward, CA, USA), according to the manufacturer’s guidance.

### 4.5. Gene Expression

The RF/6A cells were seeded in 6 well plates at a density of 1 million cells per well. The following day, the cells were treated with oxLDL and Etifoxine or XBD173 in media containing 0.1% DMSO at the same time for 24 h. The choice of the 24 h window to detect gene expression is based our previous studies [22] and a recent publication, which demonstrated that gene expression changes at 24 h in PK11195-treated cells were functionally related to tumorigenesis and apoptosis [68]. Total RNA was extracted using Tri Reagent (Sigma, UK) following the manufacturer’s instructions. The first-strand cDNA was synthesised applying the High Capacity cDNA Reverse Transcription Kits and with the RNAase inhibitor (Applied Biosystems, Birchwood, UK). The mRNA levels of the genes were then quantified using qRT-PCR assay employing a Platinum Syber Green QAPCR Super Mix-UDG w/ROX kit (Invitrogen, Inchinnam, UK). A 2^−ΔΔ*C*t^ formula was applied to quantify the relative expression of the genes which were normalised to a housekeeping gene (β-actin). The sequences of these primers used for gene expression are shown in Table 1.

### 4.6. Oil Red O (ORO) Staining

Oil Red O (Sigma, UK) was used to stain the RF/6A to detect neutral lipids. Cells were loaded with oxLDL (200 µg/mL) along with Etifoxine and XBD173 in media containing 0.1% DMSO, or the controls (media containing 0.1% DMSO), for 24 h. The cells were then stabilized with neutral buffered formalin (10%, *v*/*v*) for 1 h at room temperature. The cells were washed with PBS twice and gently rinsed with 60% isopropanol. The cells were then stained with freshly prepared 6:4 ORO with de-ionised water from the stock solution (0.5 g/mL). After 15 min of incubation, the cells were destained with 60% isopropanol briefly. The stained cells were examined under the EVOS^®^ cell imaging systems and data analysed using EVOS^®^ FL software. Additionally, the lipid droplets were resuspended with 100% isopropanol and the absorbance measured in 96well plates at 520 nm. Total cellular protein was extracted from the cells using 0.5 M NaOH and its concentration measured using the Bradford protein assay.

### 4.7. Immunocytochemistry

The RF/6A cells were seeded on a coverslip. Next day, it was incubated with a marker of functional mitochondria, MitoTracker^®^ Deep Red FM (200 nM, Thermofisher Scientific, Inchinnam, UK) in culture medium for 30 min to stain mitochondria. The cells were washed with PBS three times and fixed with 4% paraformaldehyde (*w*/*v*) and then quickly rinsed with PBS. The slides were then blocked with 2% sheep serum (*v*/*v*) and 2% BSA (*w*/*v*) in 1X PBS. Then, the slides were incubated with anti-rabbit monoclonal TSPO primary antibody (Abcam ab109497, 1:200 dilutions) for one hour, following which the slide was again blocked with 2% BSA (*w*/*v*) and then incubated with Alexa Fluor 488 secondary antibody. Subsequently, the slides were stained with DAPI (Abcam, Cambridge, UK) to stain the nucleus. The images were acquired using LSM 800 confocal microscope (Carl Zeiss Ltd., Cambridge, UK).

### 4.8. Biochemical Assays

The RF/6A cells were seeded on six-well plates and treated as above. Catalase (CAT) and Glutathione (GSSH/GSH) activities were observed in oxLDL-loaded cells treated with Etifoxine or XBD173 in media containing 0.1% DMSO and with their control (media containing 0.1% DMSO) by using OxiSelect Catalase Activity Assay Kit (Cell Biolabs, STA-341, San Diego, CA, USA) and the OxiSelect total Glutathione Assay Kit (Cell Biolabs, STA-312, San Diego, CA, USA), following the manufacturer’s guidelines. Similarly, malondialdehyde was quantified using the TBARS assay kit (Cell Biolabs, STA-330, San Diego, CA, USA) according to the manufacturer’s protocol.

### 4.9. Measurement of Cytokines

The RF/6A cells were treated with oxLDL (200 mg/mL) and Etifoxine or XBD173 in media containing 0.1% DMSO for 24 h or untreated as control (media containing 0.1% DMSO). After the incubation, the medium was collected to quantify the levels of TNF-α, IL-1β, IL6 and IL8 by Peprotech Enzyme-linked immunosorbent assay (ELISA) kits following the manufacturer’s protocols (Peprotech, London, UK).

### 4.10. Statistical Analysis

All data were collected from three independent experiments and presented as mean ± SD. The data were analysed by applying Prism 6 software using one-way ANOVA for unpaired and two-way ANOVA for all pairwise comparisons, followed by the Bonferroni test (GraphPad Software Inc., San Diego, CA, USA). *p* ≤ 0.05 was considered significant; * *p* ≤ 0.05, ** *p* < 0.01, *** *p* < 0.001 and **** *p* < 0.0001.

## 5. Conclusions

The present study demonstrates that the TSPO ligands Etifoxine and XBD-173 enhance cholesterol efflux, upregulate expression of cholesterol metabolism and transport genes, reduce oxLDL-induced ROS production, and increased antioxidant capacity and suppress inflammation in choroidal endothelial cells. TSPO ligands, particularly the clinically-used Etifoxine, have the potential to benefit AMD patients. 

## Figures and Tables

**Table 1:** Primers used for qRT-PCR.

Gene	Gene Bank Accession Number	Primer Sequences (5′–3′) Forward	Primer Sequences (5′–3′) Reverse
*β-ACTIN*	NM_001033084.1	TCCACGAAACTACCTTCAACTC	GTCATACTCCTGCTTGCTGAT
*LXRα*	XM_015114352.1	CCCCATGACCGACTGATGTT	CGGAGGCTCACCAGTTTCATT
*ABCG1*	XM_015132831.1	AAGGTGTCCTGCTACATCAT	CAGTATCTCCTTGACCATCTC
*ABCA1*	XM_015117158.1	GAAGTACATCAGAACATGGGC	GATCAAAGCCATGGCTGTAG
*CYP46A1*	XM_015144470.1	CCTTCTTCATTGCTGGTCACG	TCCATCACTGTGAACGCCAAG
*CYP27A1*	NM_001194021.1	GGCAAGTACCCAGTACGG	AGCAAATAGCTTCCAAGG
*IL-1 β*	NM_001042756.1	ACCTGAGCTCGCCAGTGAAA	GCCGGAAGCCCTCGTTGTAG
*TNF-α*	NM_001047149.1	TCTCCTTCCTGCTCGTGGCA	GGGTTTGCTACAACATGGGCTAC
*IL6*	XM_015133872.1	CCTTCCAAAGATGGCTGAAA	CAGGGGTGGTTATTGCATCT
*VEGFA*	NM_001278410.1	AAGGAGGAGGGCAGAATCAT	ATCTGCATGGTGATGTTGGA
*GPX1*	NM_001159298.2	CTCTTCGAGAAGTGCGAGGT	TCGATGTCAATGGTCTGGAA
*SOD1*	NM_001032804.1	AGGGCACCATCAATTTCGAG	ACATTGCCCAGGTCTCCAAC
*Catalase*	XM_001115625.3	CGCCTATGCAGCGAAGCTTA	TTTGCGCATCTAGCACCGGA
